# Evaluation of a novel human IgG1 anti-claudin3 antibody that specifically recognizes its aberrantly localized antigen in ovarian cancer cells and that is suitable for selective drug delivery

**DOI:** 10.18632/oncotarget.5315

**Published:** 2015-09-21

**Authors:** Chiara Romani, Emiliano Cocco, Eliana Bignotti, Daniele Moratto, Antonella Bugatti, Paola Todeschini, Elisabetta Bandiera, Renata Tassi, Laura Zanotti, Sergio Pecorelli, Enrico Sartori, Franco E. Odicino, Ario de Marco, Alessandro Davide Santin, Antonella Ravaggi, Stefania Mitola

**Affiliations:** ^1^ “Angelo Nocivelli” Institute for Molecular Medicine, Division of Gynecologic Oncology, University of Brescia, Brescia, Italy; ^2^ Department of Obstetrics, Gynecology and Reproductive Sciences, Yale University School of Medicine, New Haven, CT, USA; ^3^ Department of Molecular and Translational Medicine, Brescia, Italy; ^4^ Laboratory of Genetic Disorders of Childhood, Angelo Nocivelli Institute for Molecular Medicine, Spedali Civili, Brescia, Italy; ^5^ Department of Pathology, University of Brescia, Brescia, Italy; ^6^ Department of Biomedical Science and Engineering, University of Nova Gorica, Vipava, Slovenia

**Keywords:** claudins, tight junction, human antibody, therapeutic target

## Abstract

Membrane protein claudin3 has been recently suggested as a marker for biologically aggressive tumors and a possible target for the therapeutic delivery of active anti-cancer compounds. Claudin3-binding molecules such as the *Clostridium perfringens* enterotoxin (CPE), CPE-related molecules, and murine and chimeric antibodies have shown promising antitumor efficacy in preclinical oncological settings. We first engineered a fully human anti-claudin3 IgG1 antibody (IgGH6) by fusing the human IgG1 Fc-domain to the anti-claudin3 scFvH6 previously isolated from a pre-immune phage display library. The construct was expressed in mammalian cells and specifically targeted claudin3 endogenously expressed on the surface of different human ovarian cancer cell lines. No detectable cross-reactivity with other homologous claudins was observed. The epitope recognized by IgGH6 is located within the minor extracellular domain of claudin3 and becomes accessible only in tumor cells characterized by incomplete junction formation. Confocal microscopy experiments demonstrated that IgGH6 was actively internalized in tumor cells after binding to native claudin3 and co-localized, likely within intracellular vesicles, with the C-CPE peptide. Preliminary results indicate that IgGH6 accumulated *in vivo* in free claudin3 ovarian carcinoma xenografts. For its selective uptake in tumor cells and its human nature, IgGH6 represents a valuable candidate for antibody-drug conjugate therapeutic applications in ovarian cancer patients.

## INTRODUCTION

Claudins form a family of 24 transmembrane proteins that are major constituents of tight junction (TJ) complexes located at the apical end of the lateral surface of polarized epithelia. Although it is well established that claudin expression or subcellular localization is deregulated in a variety of human malignances [1, 2], the role of claudin in cancer progression has not been completely elucidated and seems to differ in human tumors of different origin. For example, the up-regulation of claudin3 and 4 correlates with the progression of endometrial carcinoma [3], while the reduced expression of claudin1 with poor survival in stage II of colon cancer [4]. Furthermore, claudin4 overexpression inversely correlated with the metastatic potential of pancreatic cancer cells but positively with the invasiveness of ovarian carcinoma cells [5–6].

Loss of TJ integrity leads to cell polarity impairment and increased influx of growth factors, a condition suspected to favor tumor cells survival and motility [6, 7]. In particular, claudin3 basal expression is low in normal epithelial cells, but the protein accumulates at the cell surface of several biologically aggressive human cancers, including breast, prostate, pancreatic, and epithelial ovarian tumors [8, 9]. The loss of cellular polarity and cell-cell interaction which occurs in cells during neoplastic transformation leads to the exposure of TJ components on the cell surface, making claudin3 accessible to extracellular antibody binding (“free” claudin3) [10]. Due to its differential overexpression and accessibility in a variety of human tumors, claudin3 might represent both a diagnostic biomarker and a potential therapeutic target for drug delivery.

Over the years, molecules that specifically recognize the extracellular domain of claudins have been identified and characterized. In oncological settings, the *Clostridium perfringens* enterotoxin (CPE) and the CPE-related peptides have been recently validated *in vitro* and in animal models of human cancer [11] as tumor inhibitors. CPE specifically targets the minor extracellular domain (ECL2) of claudin3 and claudin4 and strongly inhibits uterine and ovarian serous carcinoma cell growth [12]. Accordingly, Cocco et al. recently described the use of the CPE peptide as a potential carrier for the delivery of anti-tumor drugs and as an imaging agent in ovarian carcinomas [13]. Unfortunately, the non-human origin of CPE and its significant toxicity when administered systemically as full length protein will limit its use to local treatments [11–14].

Antibodies represent a valuable treatment option for the specific targeting of claudin overexpressing malignancies. A murine-human chimeric mAb against the large extracellular domain of claudin4 [15] and a chimeric dual-targeting mAb against claudin3 and claudin4 [16] have been recently developed and evaluated for their antitumor activity. These reagents demonstrated a dose-dependent ADCC on pancreatic and ovarian cancer cells supporting the anti-cancer therapeutic potential of anti-claudin antibodies. Also antibody-drug conjugates (ADC), with over 30 ADCs currently in clinical development, are valuable tools for cancer therapy. FDA approved recently two products, the CD30-targeting ADC brentuximab-vedotin for the treatment of relapsed Hodgkin lymphoma and of anaplastic large cell lymphoma [17], and ado-trastuzumab emtansine (T-DM1) for the treatment of HER2/neu-positive metastatic endometrial cancer [18].

Because of its selective accumulation in tumor cells, free claudin3 can represent an interesting cancer biomarker for targeted delivery of toxic drugs such ADCs administered systemically. This strategy is difficult to implement due to the difficulty to obtain antibodies against a specific claudin by classical immunization approaches because of the high homology of claudin sequences in human and among species. We had already isolated and characterized a human single-chain antibody (scFv) from the antibody phage display library ETH2-Gold [19], identified as scFvH6, which specifically targets the minor extracellular domain of claudin3 [20]. In this work we have first reconstituted the anti-claudin3 scFvH6 fragment into a complete fully human IgGH6 antibody, then evaluated the IgGH6 tumor-binding properties in multiple *in vitro* assays on primary ovarian and uterine cancer cell lines, and finally demonstrated its capacity to bind *in vivo* to xenograft mouse models of ovarian cancer.

## RESULTS

### Engineering, production, and validation of anti-claudin3 human IgGH6

**Figure 1:**
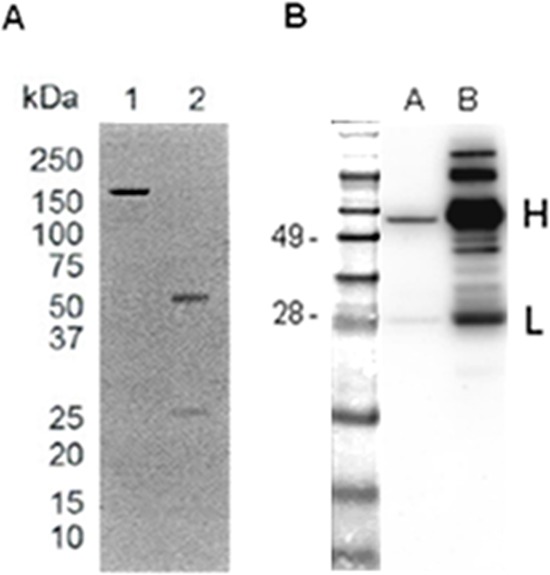
IgGH6 expression and purification evaluation IgGH6 was expressed in CHO cells and isolated by Protein A affinity chromatography. **A**. SDS-PAGE of IgGH6 under non reducing (lane 1) and reducing (lane 2) conditions. **B**. Western Blot of IgGH6. One μg of purified IgGH6 was loaded in lane A and 10 μg of human IgG was loaded as a control in lane B. Molecular weight markers are expressed in kDa.

The sequences corresponding to the VH and VL regions encoding for the scFvH6 [20] were re-cloned separately in the pFUSE-CHIg-hG1 and pFUSE2-CLIg-hk eukaryotic expression vectors for reconstituting a full-size human antibody (IgGH6). The IgG1 isotype was chosen due to its superior *in vitro* cytotoxicity and proven efficacy in clinical trials [21]. IgGH6 was produced in CHO cells to obtain antibodies with human-like post-translational modifications. Its production was assessed regularly after transfection and maximal yields were obtained after 6–7 days (data not shown). Following protein A affinity chromatography, 40 μg of purified antibody were recovered from 250 mL of culture supernatant. The correct molecular weight and IgGH6 integrity were endorsed by SDS-PAGE performed under reducing and non-reducing condition followed by Coomassie blue staining that showed that IgGH6 purity was above 95% (Figure 1A). The generation of full-size antibody was confirmed by Western Blot analysis that identified the two bands of 50 kDa and 28 kDa correspondent to the IgG H and L chains (Figure 1B). The binding capacity of the engineered IgGH6 construct towards its antigen was confirmed by surface plasmon resonance measurements. Since claudin3 is expressed at high density at the surface of ovarian tumor cells, we preferred measuring avidity rather than affinity to mimic the *in situ* actual conditions. Specifically, the avidity of the bivalent antibody for the peptide 2CL3 encompassing the claudin3 minor extracellular domain was of 15.3 nmol/L (*k*_on_ = 1.06 × 10^5^ 1/Ms; *k*_off_ = 1.62 × 10^−3^ 1/s, Supplementary Figure 1).

**Figure 2:**
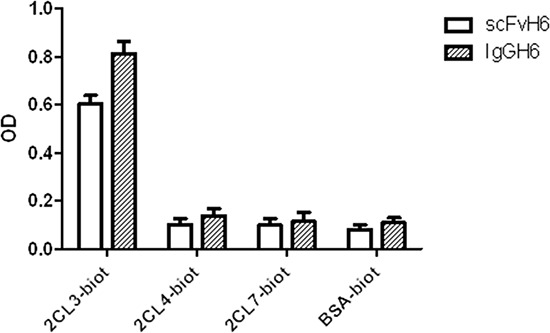
IgGH6 binds specifically claudin3 Specific binding of scFvH6 and IgGH6 to claudin3 2CL3 peptide compared to their binding to peptides 2CL4 and 2CL7 (claudin4 and 7) and to the irrelevant antigen BSA.

The specificity of IgGH6 for claudin3 was evaluated by ELISA using the peptides corresponding to the conserved minor extracellular domains of claudin3, 4, and 7 (Table 1). As shown in Figure 2, IgGH6 retained the target specificity encoded by the original scFvH6 and exhibited high selectivity towards the desired antigen with negligible cross-reactivity to homologous claudins [22].

**Table 1:** Amino acid sequences of the peptides corresponding to the minor extracellular domains of claudin3, claudin4, and claudin7

peptide	amino acid sequence	claudin subtype
2CL3	PVSWSANTIIRDFYNPVVPEAQKREMGAGLY	3
2CL4	TAHNIIQDFYNPLVASGQKREM	4
2CL7	WYGHQIVTDFYNPLIPTNIKYE	7

### Binding properties on cancer cells

**Figure 3:**
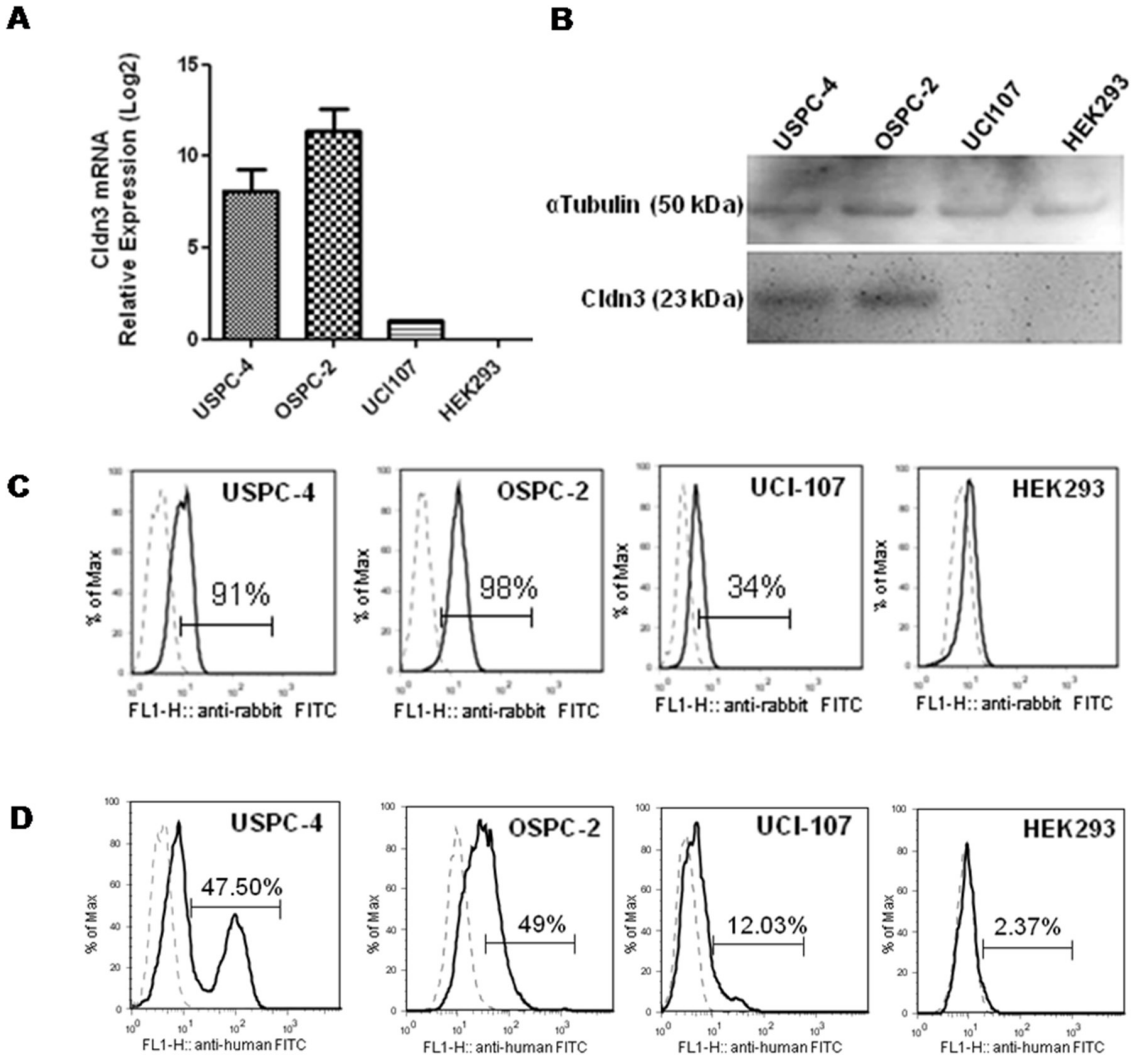
IgGH6 binding properties on free claudin3 expressing human cancer cell lines **A.** Expression of claudin3 mRNA in human cancer cell lines USPC-4, OSPC-2, UCI-107 and normal human embryonal kidney HEK293 cells was measured by RT-qPCR analysis. Data are shown as the mean ± SE of two independent experiments and are expressed as relative expression ratios (ΔΔCt – Fold increase) using HEK293 as a reference. **B.** 20 ug of cell lysates were separated by SDS-PAGE and probed with anti-claudin3 polyclonal antibody. Equal loading was confirmed by incubating the membrane with αTubulin. **C.** Total claudin3 expression level was evaluated by FACS analysis on fixed and permeabilized cells. Single cell suspension were incubated with commercial antibody against claudin3 followed by incubation with anti-rabbit-FITC conjugated antibody. Histograms of cells stained with an isotype control antibody (dotted line) or with specific anti-claudin3 (solid line) are shown for each cell line tested. The percentage of claudin3 positive cells is reported in each plot. **D.** IgGH6 binding to the extracellular domain of claudin3 was evaluated by FACS analysis on unfixed cells. Single cell suspensions were incubated with 2.5 μg/mL of purified IgGH6 for 1 h followed by a 30 min incubation with mouse anti-human IgG-FITC secondary antibody, and analyzed by FACS gating on annexin V negative cells. Histograms of cells stained with specific IgGH6 (solid line) or only secondary antibody (dotted line) are shown for each of the cell lines tested. The percentage of claudin3 positive cells is reported in each plot.

The binding proprieties of IgGH6 on cells were evaluated using cancer cell lines expressing variable amounts of claudin3. To this aim, different primary ovarian and uterine serous carcinoma cells were tested for claudin3 expression by RT-PCR, FACS analysis and Western blotting using commercial rabbit polyclonal antibodies against the C-terminus intracellular region of claudin3. RT-PCR allowed for the identification of tumor cell lines with relative high (OSPC-2), intermediate (USPC-4), and low (UCI-107) expression of claudin3 (mean claudin3 expression was 11.4 folds and 8.1 folds higher in OSPC-2 and USPC-4 than in UCI-107) (Figure 3A). Western blot analysis confirmed at protein level (Figure 3B) that claudin3 accumulated in USPC-4 and OSPC-2 cells at higher levels than in UCI-107, whereas no evidence of claudin3 presence was observed in TJ-free HEK293 control cell line. Similar results were obtained by FACS analysis using the commercial antibody. Specifically, 91–98% of USPC-4 and OSPC-2 cells expressed claudin3 with a MFI of 5.87 and 10.54 on positive cells respectively, while only 34% of UCI-107 expressed claudin3 with a MFI of 2.43 (Figure 3C). These cell lines were gently detached with EDTA to preserve membrane integrity and avoid false positivity due to mechanical damage before being used to assess the IgGH6 ability to bind the extracellular domain of claudin3. Cells were incubated 1 hour with 2.5 μg/mL of purified IgGH6 followed by 30 min incubation with mouse anti-human IgG-FITC secondary antibody before FACS analysis. Cells with damaged membrane labeled with Annexin V were excluded from the analysis. Figure 3D shows that 47%, 49% and 12% of USPC-2, OSPC-4, and UCI-107 cells, respectively, were recognized by IgGH6. The mean of fluorescence of USPC-4, OSPC-2 and UCI-107 was 53, 43 and 16, respectively, consistent with the different claudin3 accumulation at the surface of these cells. HEK293 cells with no detectable claudin3 accumulation served as a negative control and did not show any significant shift in FACS histogram.

### Cellular fate of IgGH6 after binding to claudin3 on the cell membrane

**Figure 4:**
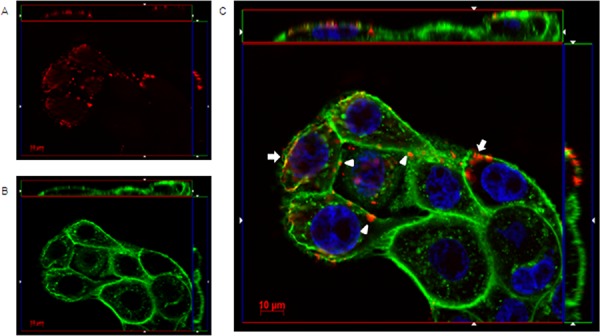
IgGH6 binds claudin3 on USPC-4 cell surface USPC4 cells were incubated 1 h at 4°C with 2.5 μg/mL of IgGH6 to prevent antibody internalization, then cells were fixed, permeabilized, and incubated with the Alexa594-conjugated secondary antibody (red) **panel A.** Cell membrane and nuclei were stained with WGA-Alexa488 (green) **panel B** and DAPI (blue), respectively. Images were collected using a Zeiss Axiovert 200M epifluorescence microscope equipped with a Plan-Apochromat 63x/1.4 NA oil objective; z-stack images were acquired using ApoTome system and elaborated Inside4D module. **panel C** shows images resulting from merging the three different channels. IgGH6 binding at the cell-cell contact (arrowhead) and on the cell surface outside the TJ area (arrow) are visualized.

Immunofluoresence microscopy was used to visualize the interaction mechanisms between IgGH6 and claudin3 on cell membranes. When USPC-4 cells were incubated at 4°C to prevent receptor internalization, IgGH6 localized at the cell surface, as demonstrated by the specific staining that was restricted to foci present along the whole cell margins (Figure 4). The pictures clearly indicate that the accumulation of claudin3 in OSPC-2 is not localized in cell-cell contact regions, as typically observed for TJ proteins as claudins in healthy tissues, but appears also in the upper portion of the cell membrane, proving the mislocalization of claudin3 in ovarian tumor cells [23].

**Figure 5:**
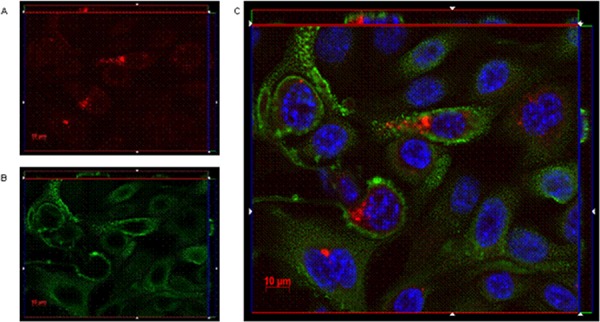
IgGH6 binds and internalizes claudin3 in OSPC-2 cells OSPC-2 cells were incubated 1 h at 37°C with 2.5 μg/mL of IgGH6 to allow antibody internalization, then cells were fixed, permeabilized and incubated with the Alexa594-conjugated secondary antibody (red) **panel A.** Cell membrane and nuclei were stained with WGA-Alexa488 (green) **panel B.** and DAPI (blue), respectively. Images were collected using a Zeiss Axiovert 200M epifluorescence microscope equipped with a Plan-Apochromat 63x/1.4 NA oil objective; z-stack images were acquired using ApoTome system and elaborated Inside4D module. **panel C.** shows images resulting from merging the three different channels.

**Figure 6:**
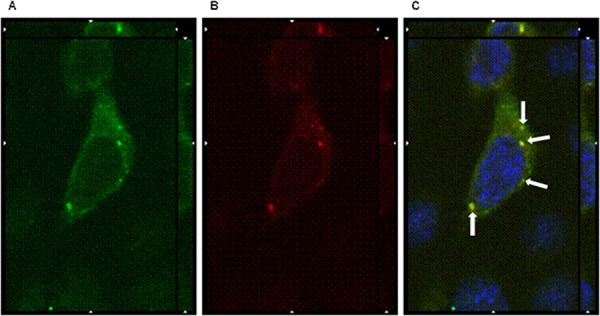
IgGH6 internalizes with CPE_290–319_ peptide OSPC-2 cells were incubated 1 h at 37°C with 2.5 μg/mL of IgGH6 in the presence of [10 μg/mL] FITC-CPE_290–319_ peptide (green) **panel A** to allow antibody and peptide internalization, then cells were fixed, permeabilized and incubated with the Alexa594-conjugated secondary antibody (red) **panel B.** Nuclei were stained with DAPI (blue). Images were collected using a Zeiss Axiovert 200M epifluorescence microscope equipped with a Plan-Apochromat 63x/1.4 NA oil objective; z-stack images were acquired using ApoTome system and elaborated Inside4D module. **panel C** shows images resulting from merging the three different channels, with arrows showing IgGH6 and CPE peptide colocalization in the cell cytoplasm.

When cells were incubated 1 hour at 37°C, the orthogonal reconstruction of images relative to OSPC-2 (Figure 5) and USPC-4 (Supplementary Figure 2) reveals intense intracellular fluorescence signal localized in spots resembling endosomal vesicles. The carboxy-terminal fragment (C-CPE_290–319_ peptide) of *Clostridium perfringens* enterotoxin is known to gradually internalize into the cytoplasm of claudin3 and claudin4 positive ovarian cancer cells after binding to the minor extracellular domain of the claudins [13]. We hypothesized a similar mechanism for the IgGH6 uptake and, consequently, incubated 1 hour at 37°C OSPC-2 cells in the presence of both FITC conjugated C-CPE_290–319_ peptide and IgGH6. As shown in Figure 6, the two claudin3 binders show a strong co-localization inside the cells suggesting that the antibody undergoes the same claudin3-mediate internalization process previously described for the C-CPE peptide [14].

### IgGH6 *in vivo* specific targeting of free claudin3 tumors

**Figure 7:**
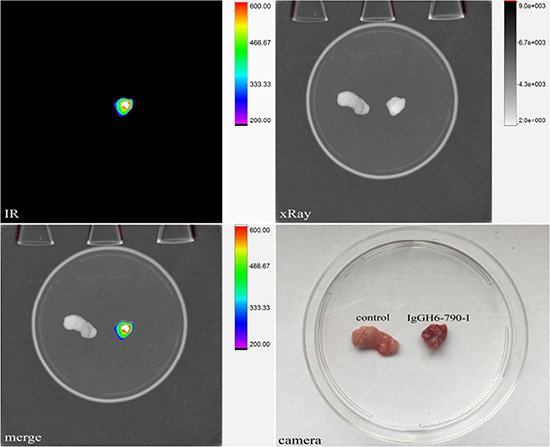
IgGH6-790-I tumor binding capacity *in vivo* OSPC-ARK-1-derived xenografts were injected IV with 20 μg of IgGH6-790-I. After 6 hours, mice were sacrificed and tumors were excised and visualized using an *In-Vivo* FX PRO system (Bruker Corporation, Billerica, MA; excitation/emission 760/830 nm; exposure time 60 seconds). Strong fluorescence was observed in tumors excised from IgGH6-790-I injected animals while negligible staining was detected in tumors excised from mice injected with the vehicle (PBS).

To assess whether IgGH6 was able to localize to free claudin3-overexpressing ovarian cancer *in vivo*, sub-cutaneous OSPC-ARK-1-derived xenografts were generated as previously described [24]. Four weeks after tumor implantation, 20 μg of IgGH6 labeled with the Near InfraRed dye 790-I were injected intravenously and 6 hours later tumors were excised and visualized using an *In Vivo* FX PRO system. Strong fluorescent signal was observed in tumors excised from animals injected with IgGH6-790-I, while negligible staining was detected in tumors excised from vehicle-injected control mice (Figure 7, IR, merge and camera pictures).

## DISCUSSION

The possibility of targeting specifically neoplastic cells while sparing healthy tissues would significantly improve the tollerability of anti-cancer therapies and, accordingly, there is an enormous interest in identifying biomarkers and reagents suitable for selective drug delivery. Claudin3 has recently emerged as a potential cancer biomarker as a diagnostic as well as a therapeutic target [8, 12] because it is over-expressed selectively in several biologically aggressive human tumors. Our group has already described the feasibility to target aggressive gynecologic cancers with claudin3- and 4-binding CPE [13, 25]. The molecular mechanism of the CPE-claudin3 interaction has been extensively investigated and allowed for the identification of the minor extracellular domain as the sequence motif involved in the CPE binding [26]. Unfortunately, the CPE toxicity prevents its systemic administration in human patients, whereas the development of novel and nontoxic analogues specific for claudin3 would be highly desirable.

Recently, our group isolated a recombinant antibody fragment (scFvH6) specific to the ECL2 domain of claudin3 using a synthetic human phage display library [20]. Given the promising results obtained with anti-claudin3 murine and chimeric antibodies used for ADCC [15, 16], we developed a full human functional IgG reagent (IgGH6) starting from the original scFvH6 fragment molecule that conserved the original selective binding characteristics for the claudin3 expressed at the surface of ovarian cancer cells.

Extracellular domains of claudins are involved in the formation of TJ, in which they interact in homotypic and heterotypic manner [27]. Whereas in normal confluent epithelial cell monolayer claudin3 is expected to localize within the TJ strand at the apical site of the lateral membrane, the known dis-regulation of the mitotic spindles during epithelial tumorigenesis and the resulting out-of-plane division in tumor cells was suspected to induce abnormal localization of TJ components at the cell surface [28]. However, the detection of “free” claudins, and specifically of claudin3, in such tumor cells has been difficult to assess in the past because of the lack of commercially available antibodies specific for claudin extracellular domains. In the present study, the IgGH6-immunofluorescence of USPC-4 and OSPC-2 cancer cell lines clearly demonstrated scattered focal claudin3 expression along the whole cell membrane, instead of the physiological distribution pattern of healthy cells in which the protein is strictly localized at TJ. Our results are in agreement with those of Winkler et al. [26] who demonstrated that CPE binds to the free domain of claudin3 that is not occupied by trans-interaction with homologous claudins and is not incorporated in TJ strands. Summarizing, the collected data underline two issues that are critical for the therapeutic potential of IgGH6: i) in cancer cell lines this fully human antibody reacted exclusively with claudin3 not engaged in forming TJ; ii) this form of free claudin is a specific biomarker for ovarian cancer cells.

In principle, the focal expression of the target antigen on cancer cell surface may diminish the anti-tumor efficacy of an antibody designed for ADCC therapy because of the limited number of targetable molecules. However, the very specific accumulation of the IgGH6-targetable, accessible free claudin3 only in cancer cells and its absence in healthy, polarized epithelial cells would represent ultimately a major advantage for ADC [29] because selectivity for the tumor cells and minimal toxicity are the most relevant requisites for this therapeutic approach. Also the confocal microscopy experiments that demonstrated the fast and effective internalization of IgGH6 in OSPC-2 and USPC-4 cells favor the use of this antibody for ADC [30, 31] rather than for ADCC that requires the stable binding of the antibody to the external surface of the target cells. Remarkably we have also shown that IgGH6 accumulates *in vivo* in xenograft models of free claudin3-expressing ovarian cancer following systemic administration. Tumor explanted 6 hrs after IgGH6-790-I injection was strongly fluorescent, while untreated tumor tissues did not show any detectable signal.

In contrast to the already available anti-claudin murine and chimeric antibodies [15, 16], the human nature of IgGH6 would minimize immune reactions that could prevent the treatment iteration. To our knowledge, this is the first human antibody which specifically targets the minor extracellular domain of the tumor-associated claudin3.

Now that the binding and interalization kinetics of IgGH6 have been thorughly characterized, we plan its fusion to active toxins and the *in vivo* experiments necessary to identify the optimal treatment conditions and to validate the therapeutic potential of IgGH6-based ADC reagents to use against claudin3-expressing malignancies.

## MATERIALS AND METHODS

### Cancer cell lines and cell culture

Primary ovarian serous carcinoma cell line OSPC-2 and chemotherapy-resistant primary ovarian serous papillary carcinoma cell line OSPC-ARK1 were established from samples obtained, respectively, at the time of primary surgery or collected at the time of tumor recurrence from a patient harboring stage IV OSPC. Primary uterine serous carcinoma cell line USPC-4 was established from samples collected at the time of tumor recurrence from an intra-abdominal metastatic site. Primary cell lines and ovarian carcinoma cell line UCI-107 were maintained at 37°C in 5% CO_2_ in RPMI-1640 medium supplemented with 10% FBS. HEK293 cells, free of endogenous claudins and of TJ strands [32] were evaluated as a negative control. HEK293 were cultured in DMEM (Dulbecco's modified Eagle's medium) supplemented with 10% FBS. Chinese hamster ovary (CHO) cells, cultured in suspension (1 × 10^5^ cells/mL) in CD-CHO medium (GIBCO, Life Technologies) supplemented with L-Glutamine 8 mM and HT Supplement, were incubated at 37°C in a humidified atmosphere of 5% CO_2_ on an orbital shaker platform.

### Quantification of claudin3 mRNA by real-time PCR

Total RNA was extracted with Trizol reagent (Life Technologies) and treated with TURBO DNase enzyme (Ambion, Applied Biosystems) to remove the contaminating DNA eventually present. First-strand cDNA was synthesized using SuperScript II reverse transcriptase (Life Technologies) and q-PCR performed with an ABI Prism 7000 Sequence Analyzer according to the manufacturer's instructions (Applied Biosystems). 25 ng of cDNA were amplified by using the TaqMan Universal PCR Master Mix (Applied Biosystems). The comparative threshold cycle (Ct) method was used to determine gene expression in each sample relative to the value observed in HEK293 cells. The mRNA expression levels of target genes were normalized to the levels of GAPDH transcript. The primers for claudin3 were obtained from Applied Biosystems as Assay-on-Demand products. Assay ID were Hs00265816_s1 (claudin3) and Hs99999905_m1 (GAPDH).

### Cloning of IgGH6 into eukaryotic expression vector and transfection in CHO cells

The VH region encoding scFvH6 was PCR-amplified using the reverse primer H6-VH-Rev (GCTAGCACTCGAGACGGTGACCAGGGTTCC, corresponding to downstream VH sequence, with the underlined internal *NheI* restriction enzyme site) and the forward primer IL2-H6-VH-Fw (CTCGAGatgtacaggatgcaactcctgtcttgcattgcactaagtcttgcac ttgtcacgaattcgGAGGTGCAGCTGTTGGAGTCT), containing the IL2 signal peptide sequence (lowercase) in frame with the upstream VH sequence (uppercase) and an internal *XhoI* restriction enzyme site (underlined). The PCR product was digested with *NheI* and *XhoI* before being inserted into the expression vector pFUSE-CHIg-hG1 (InvivoGen), featuring the constant region of the human IgG1 heavy chain, predigested with the same enzymes.

The VL scFvH6 domain was PCR amplified using the reverse primer H6-VL-Rev (CGTACGTTTGATTT CCACCTTGGTCCCTTG, corresponding to downstream VL sequence, with the underlined internal *BsiWI* restriction enzyme site) and the forward primer IL2-H6-VL-Fw (ACCGGTatgtacaggatgcaactcctgtcttgcattgcactaagtcttg cacttgtcacgaattcgGAAATTGTGTTGACGCAGTCT containing the IL2 signal peptide sequence (lowercase) in frame with the upstream VL sequence (uppercase) and an internal *AgeI* restriction enzyme site (underlined). The VL PCR product was digested with *BsiWI* and *AgeI* and cloned into the expression vector pFUSE2-CLIg-hk (InvivoGen), featuring the constant region of the human immunoglobulin kappa light chain, predigested with the same enzymes. The two final resulting constructs, named pFUSE-H6VH and pFUSE-H6VL, were then expressed in CHO cells and secreted into the medium as a complete IgG. Lipofectamine2000 (Life Technologies) was used to transiently transfect 50 μg of pFUSE-H6VH and 50 μg of pFUSE-H6VL into 4 × 10^7^ CHO cells re-suspended in 30 mL of CD-CHO serum-free medium in a 125 mL spinner flask. Cells were cultured at 37°C on an orbital shaker with humidified atmosphere containing 5% CO_2_. The medium was collected the third day after cell centrifugation, replaced by fresh medium and collected again after further 4 days of culture.

### Production of human IgGH6

ELISA was used to verify the presence of IgGH6 in CHO supernatant. Streptavidin-coated plates were incubated o/n at 4°C with biotin conjugate-goat-anti-human IgG γ chain specific (1:10000 in PBS, Sigma Chemical Company). After blocking, 100μL of IgG-containing supernatant were added to each well and incubated for 1.5 h at RT. Bound IgGs were coupled to mouse-anti-human IgG κ chain specific (Santa Cruz, 1:500 in 1% milk/PBS) and anti-mouse HRP-conjugated antibody (Sigma Chemical Company). Color reaction was performed in the presence of soluble BM blue POD substrate (Roche Diagnostics) and was stopped by the addition of 1 M sulfuric acid before plate reading at 450nm. IgGH6 was purified from CHO supernatants using Protein A. Briefly, supernantant was loaded onto a 1-mL Protein A Sepharose Fast Flow resin packed in a chromatography column (BioRad Laboratories). The column was extensively washed with 20 mM sodium phosphate buffer, pH 9. Bound antibodies were eluted with 0.1 M citric acid, neutralized with 100 mM Tris-HCl, pH9, and concentrated on Amicon-Ultra 10K cartridges (Amicon). Protein A-purified IgGH6 fractions were separated by SDS-PAGE and visualized by Coomassie blue staining. IgGH6 heavy and light chains were simultaneously detected in a Western Blot assay with a biotin-conjugated polyclonal antibody against human IgG (Jackson ImmunoResearch Laboratories) coupled with HRP-conjugated streptavidin, and immunoreactions developed by a chemiluminescent substrate (ECL; Amersham Biosciences Corp.).

### ELISA binding assay

The 31 amino acid peptide corresponds to the second extracellular loop of claudin3 (2CL3) according to the predictive model of claudin3 [33] and was previously used for the scFvH6 isolation from a pre-immune library [20]. The same peptide was used in combination with the two peptides 2CL4 and 2CL7 corresponding to the second extracellular domain of claudin4 and claudin7 (Table 1) to test the IgGH6binding specificity by ELISA. *In vitro* peptide synthesis and N-biotinylation was performed at Alpha Diagnostics International. The peptides (100 ng/well) were absorbed on a streptavidin-coated 96 well plate overnight at 4°C. After blocking (5% milk), 100 μL of IgGH6-containing supernatant or 400 ng/well of ScFvH6 were added and incubated for 1 h at RT. Bound IgGH6 was detected with goat anti-human Fc HRP-conjugated antibody (1:10.000; Sigma Chemical Company). Bound ScFvH6 was detected with an anti-myc tag antibody (9E10 clone,1:1500; Roche Diagnostics), followed by anti-mouse HRP-conjugated antibody (Sigma Chemical Company) as previously described [20]. The immunoreactions was developed with soluble BM blue POD substrate (Roche Diagnostics), stopped by the addition of 1 M sulfuric acid before plate reading at 450nm.

### Surface plasmon resonance assay

SPR measurements were performed on a BIAcore X instrument (GE-Healthcare Life Science). Biotinylated 2CL3 peptide (2 μg/mL in 10 mM HEPES, pH 7.4, 150 mM NaCl, 3 mM EDTA, 0.005% surfactant P20) was coated onto the SA streptavidin sensor chip at an immobilization density of 1,612 resonance units (RU) equal to 0.35 pmol/mm^2^ of the peptide. Increasing concentrations of IgGH6 in the same buffer buffer were injected 4 min before dissociation measurement (10 min). After every run, the sensor chip was regenerated by the injection of 10 mM glycine, pH 2.0.

### Flow cytometry

Total claudin3 expression was evaluated on permeabilized cells with rabbit anti-claudin3 polyclonal antibody (Life Technologies), followed by incubation with anti-rabbit-FITC secondary antibody. The ability of IgGH6 to recognize cell surface claudin3 was tested on unfixed OSPC-2, USPC-4, UCI-107, and HEK293 cells. After detachment with 0.5 mM EDTA, cells were incubated for 1 h with 2.5 μg/mL of purified IgGH6 followed by a 30 min incubation with mouse anti-human IgG-FITC secondary antibody (Southern Biotech). Cells were acquired on a FACS-Calibur flow cytometer and samples were analyzed by Cell quest Pro Software (Becton Dickinson) gating on Annexin V (BD Pharmingen) negative cells. Percentages and mean fluorescence intensity (MFI) of positive cells were determined considering the position of hystograms of unstained cells and subtracting MFI values of their natural fluorescence, respectively.

### Immunofluorescence staining and internalization

OSPC-2, USPC-4 and UCI-107 cells were seeded on cell culture slide (SPL Life Science Co), grown to 50 to 70% confluency and treated with 2.5 μg/mL of IgGH6 for 1 hour at 4°C or at 37°C in the absence or in the presence of FITC-conjugated *Clostridium perfringens* carboxy-terminal fragment CPE_290–319_ peptide (10 μg/mL). After washing, cells were fixed with 4% formaldehyde for 15 min at RT and incubated with 0.5 μg/mL of anti-human IgG-Alexa647-conjugated antibody. Membrane and nuclei were stained with WGA-Alexa488 (Life Technologies) and DAPI respectively. Finally, cells were washed with PBS and mounted in ProLong Gold antifade reagent (Life Technologies). Samples were analyzed using an epifluorescence microscope Axiovert 200 equipped with a 63x/1.4 NA Oil Objective and APOTOME.2 system. Serie of Z stack fluorescent images were analyzed by INSIDE 4D and Extended focus modules of Axiovision software (CarlZeiss).

### IgGH6-790-I generation and *in vivo* tumor binding capacity

The labeling of IgGH6 to the NearInfraRed dye 790-I was performed using a commercially available kit according to the manufacturer's protocol (Abnova, #KA4185). For the *in vivo* studies, sub-cutaneous OSPC-ARK-1-derived xenografts were generated as previously described [24]. Briefly, C.B-17/SCID female mice 5–7 weeks old were purchased from Harlan Sprague-Dawley (Indianapolis, IN) and housed in a pathogen-free environment at Yale University. They were given basal diet and water ad libitum. All experimental procedures were approved by the Institutional Animal Care and Use Committee (IACUC). C.B-17/SCID mice were injected subcutaneously with 5 × 10^6^ cells derived from OSPC-ARK-1. Four weeks later, 20 μg of IgGH6-790-I were injected IV and 6 hours later animals were sacrificed and tumors were excised and visualized using an *In-Vivo* FX PRO system (Bruker Corporation, Billerica, MA; excitation/emission 760/830nm; exposure time 60 seconds). As controls, tumors excised from mice injected with the vehicle solution were visualized using the same protocol.

## SUPPLEMENTARY FIGURES


