# Supplementary material for: Evaluation of a novel human IgG1 anti-claudin3 antibody that specifically recognizes its aberrantly localized antigen in ovarian cancer cells and that is suitable for selective drug delivery
Source: Oncotarget. 2015 Sep 21;6(33):34617–28. doi: 10.18632/oncotarget.5315 (PMC4741477; doi:10.18632/oncotarget.5315)
Supplement: Supplementary file 1 [file oncotarget-06-34617-s001.pdf]

SUPPLEMENTARY FIGURES

A

Sample	K_{on} ($M^{-1}s^{-1}$)	K_{off} (s^{-1})	Kd (mol/L)
IgGH6	1.06E+05	1.62E-03	15.3 nmol/L

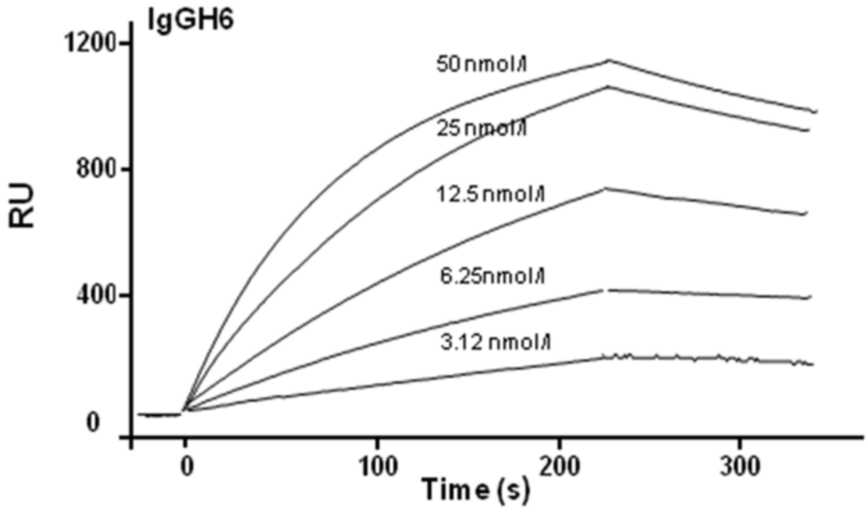

B

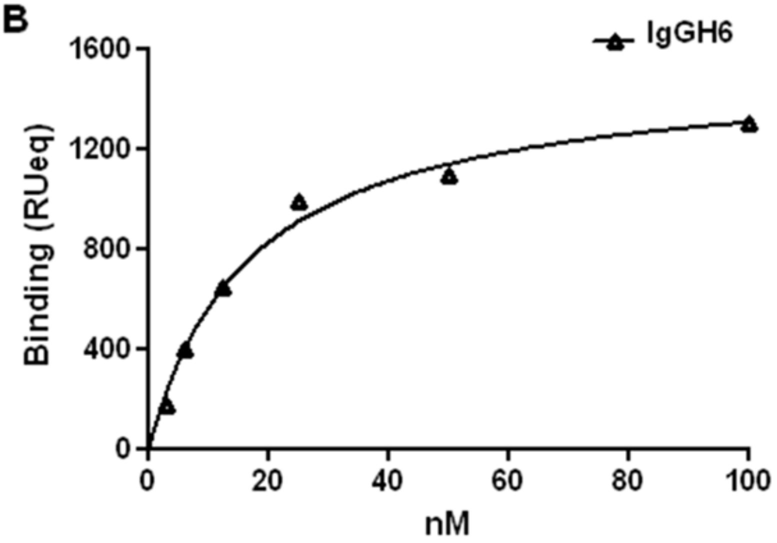

Supplementary Figure S1: IgGH6 binding kinetics. A. Increasing concentrations of IgGH6 were injected on an 2CL3-coated SA sensor chip. Response units (RU) were recorded as the function of time. An overlay plot is shown for all sensorgrams after subtraction of their corresponding control sensorgrams. **B.** Scatchard plot analysis of the equilibrium binding data.

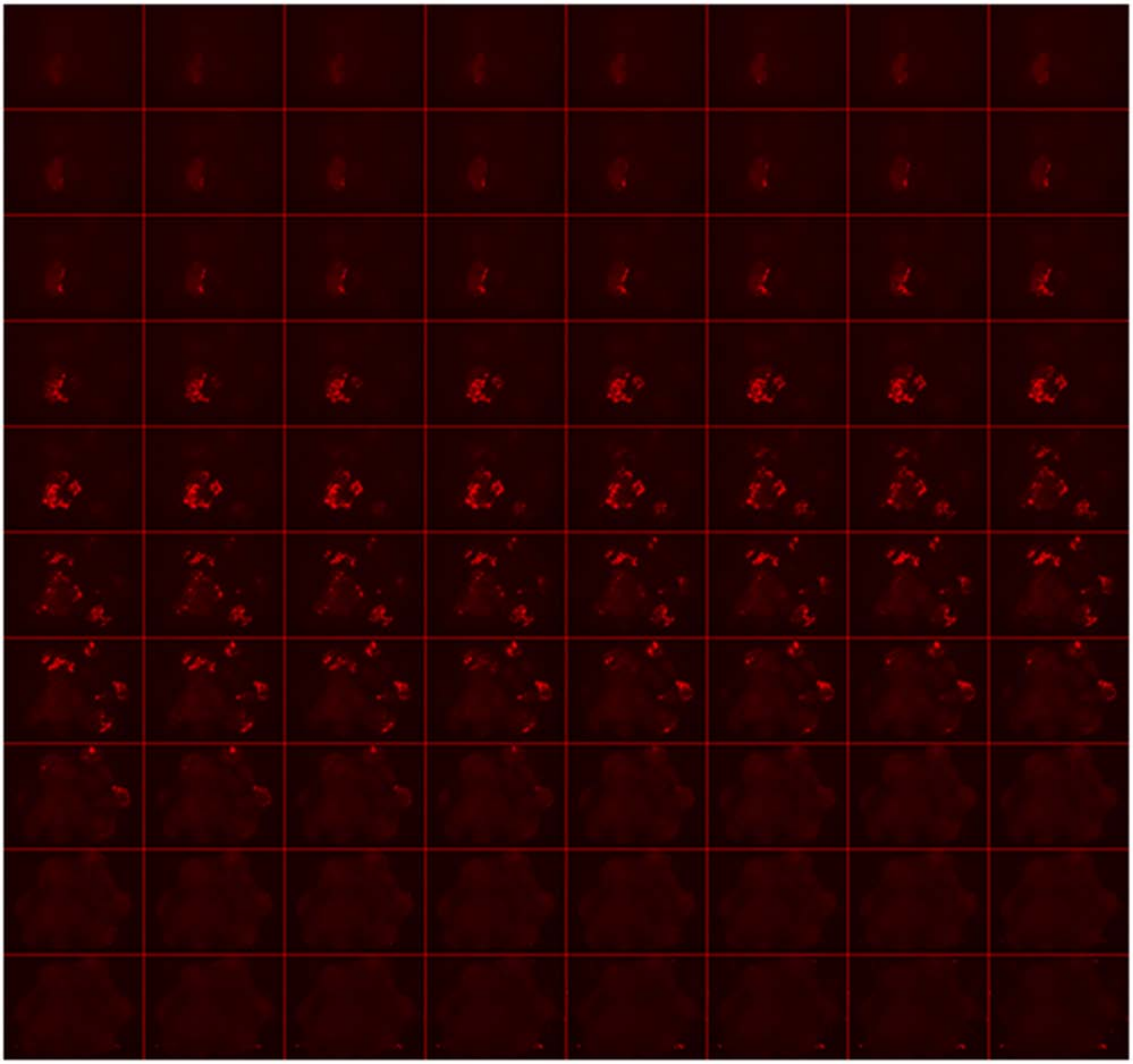

Supplementary Figure S2: Confocal microscopy z-stack analysis of intracellular distribution of IgGH6 in USPC-4 cells. Images were reconstructed from confocal micrographs taken every 0.25 μm from the bottom of the culture.
